# Supplementary material for: BAF complexes drive proliferation and block myogenic differentiation in fusion-positive rhabdomyosarcoma
Source: Nat Commun. 2021 Nov 26;12:6924. doi: 10.1038/s41467-021-27176-w (PMC8626462; doi:10.1038/s41467-021-27176-w)
Supplement: Supplementary file 1 — Supplemental Information [file 41467_2021_27176_MOESM1_ESM.pdf]

BAF complexes drive proliferation and block myogenic differentiation in fusion-positive rhabdomyosarcoma

Keywords: Chromatin remodelers, BAF, Rhabdomyosarcoma, Epigenetics, BRG1, myogenesis, MYCN

Author List and affiliations

Dominik Laubscher[1*], Berkley E. Gryder[2,3*], Benjamin D. Sunkel[4*], Thorkell Andresson[5], Marco Wachtel[1], Sudipto Das[5], Bernd Roschitzki[6], Witold Wolski[6], Xiaoli S. Wu[7], Hsien-Chao Chou[2], Young K. Song[2], Chaoyu Wang[2], Jun S. Wei[2], Meng Wang[4], Xinyu Wen[2], Quy Ai Ngo[1], Joana G. Marques[1], Christopher R. Vakoc[7], Beat W. Schäfer[1†], Benjamin Z. Stanton[4,8,9†], and Javed Khan[2†]

1- Department of Oncology and Children's Research Center, University Children's Hospital, Zurich, Switzerland

2- Genetics Branch, NCI, NIH, Bethesda, MD, USA

3- Department of Genetics and Genome Sciences, Case Western Reserve University, Cleveland, OH, USA

4- Nationwide Children's Hospital, Center for Childhood Cancer and Blood Diseases, Columbus, OH, USA.

5- Protein Characterization Laboratory, Cancer Research Technology Program, Frederick National Laboratory for Cancer Research, Frederick, MD

6- Functional Genomics Center, University of Zurich/ETH Zurich, Zurich, Switzerland

7- Cold Spring Harbor Laboratory, 1 Bungtown Road, Cold Spring Harbor, NY 11724, USA

8- Department of Pediatrics, The Ohio State University College of Medicine, Columbus, OH, USA

9- Department of Biological Chemistry & Pharmacology, The Ohio State University College of Medicine, Columbus, OH, USA.

Additional Information

* These authors contributed equally

[†]These authors jointly supervised this work

Benjamin Z. Stanton, Ph.D. Benjamin.Stanton@nationwidechildrens.org

Beat Schäfer, Ph.D. Beat.Schaefer@kispi.uzh.ch

Javed Khan, M.D. khanjav@mail.nih.gov

All authors declare no conflicts of interest.

Supplementary Figures and Legends

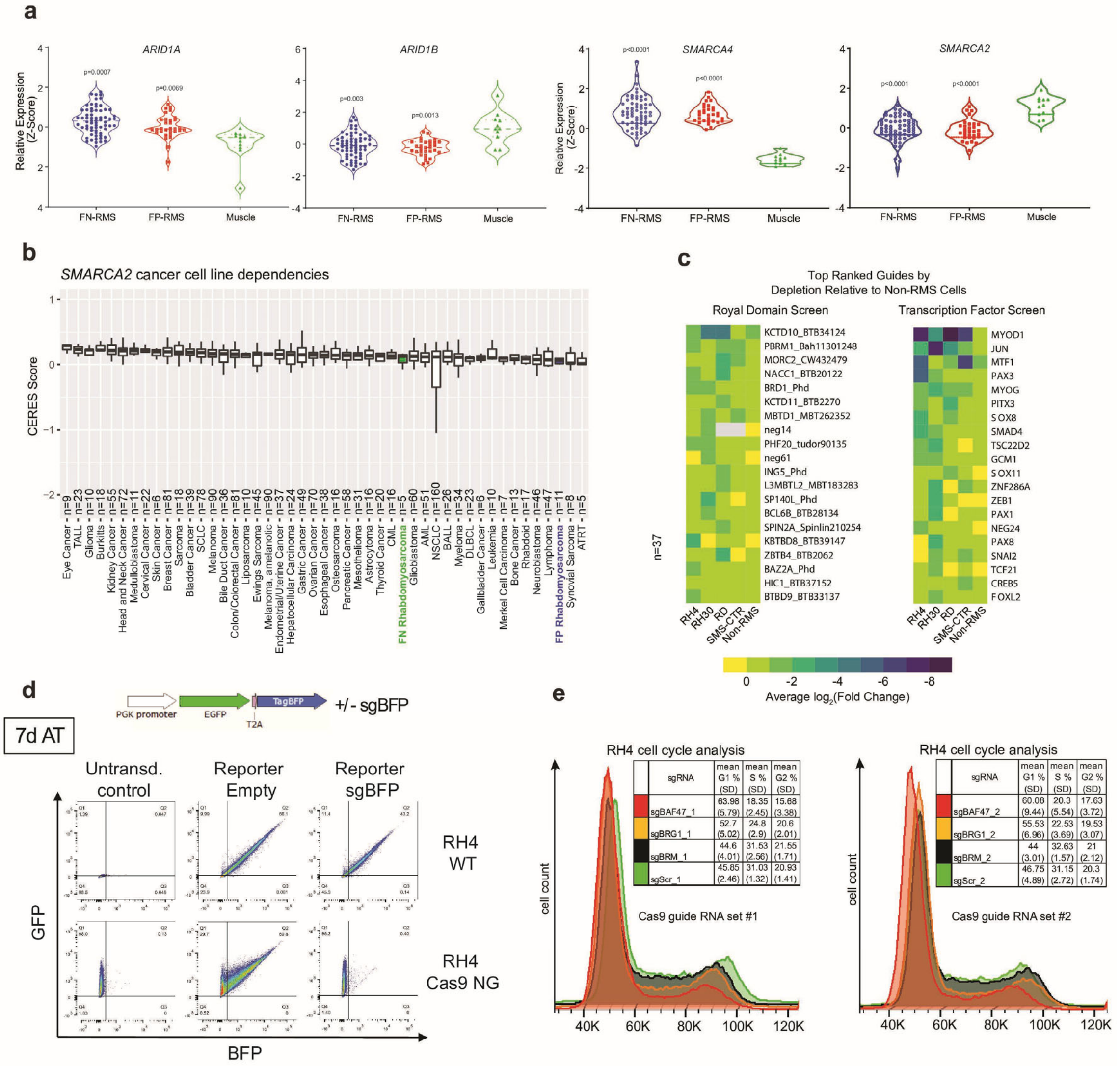

Figure S1. BRG1 containing BAF complexes are important for FP-RMS tumor maintenance. (**a**) Relative expression levels of indicated genes represented as Z-Scores between FN-RMS (n=67) and FP-RMS (n=31) tumors compared to normal muscle tissue (n=11). Statistical significance is indicated based on unpaired t-tests with Welch's correction (two-tailed). Accession details for public gene expression datasets (phs000720) can be found in the data availability section. (**b**) Data from DepMap was analyzed to determine the relative dependence of RMS cells on BRM (encoded by *SMARCA2*) compared to other cancer cell line disease types (n=sample number). Box plots of median and quartiles, with whiskers showing 1.5 × inter-quartile ranges. (**c**) CRISPR screens targeting chromatin regulatory reader domains and transcription factors were performed in FP-RSM cells (RH4 and RH30), FN-RMS cells (RD and SMS-CTR) cells, and non-RMS control cell lines. Fold depletion values in all cell types were directly compared and guides were ranked by specific depletion in RMS vs. non-RMS cells. (**d**) Activity of Cas9 in engineered RH4 cells measured in BFP reporter assay. WT or Cas9 expressing cells were transduced with either empty or BFP targeting sgRNA containing reporter constructs. Fluorescence was measured 7 days after transduction. Depletion of BFP positive population is nearly complete in Cas9 expressing cells while in WT cells this population is stable. (**e**) Representative cell cycle histogram profiles of Cas9 expressing RH4 cells 7 days after transduction with indicated sgRNAs determined by PI staining measured by flow cytometry. Insert tables represent cell cycle distributions determined by FlowJo v10 software using the Dean-Jett-Fox algorithm.

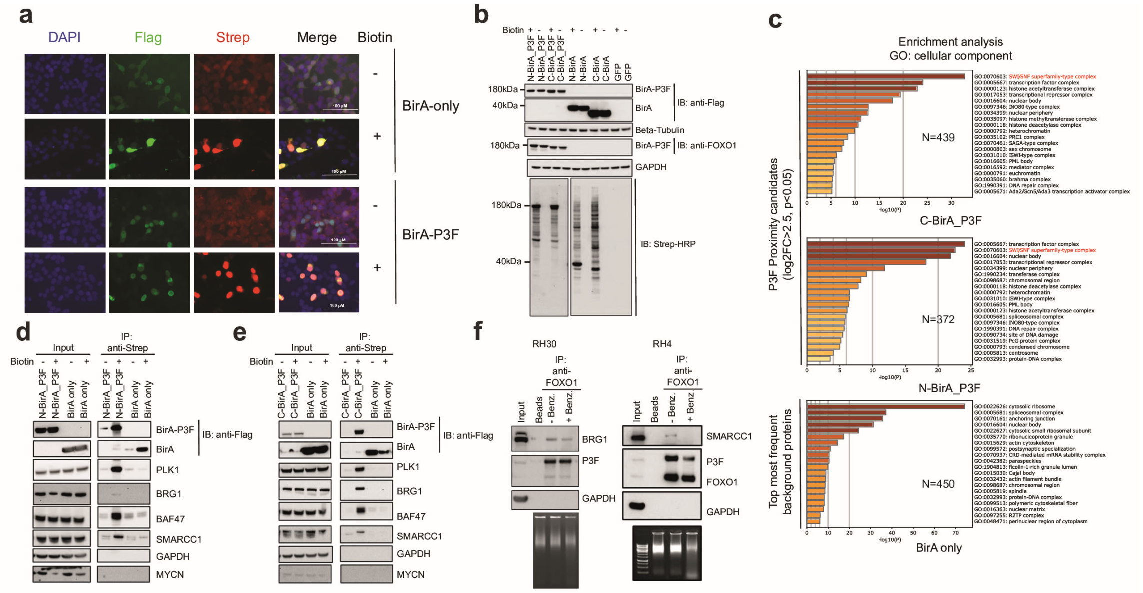

Figure S2. Proximity and Interaction studies by BioID and CoIP. (**a**) Immunofluorescence pictures for HEK293T cells 24h after transfection with indicated constructs either with or without biotin addition. Flag staining corresponds to BirA only or BirA/PAX3-FOXO1 localization. Strep staining corresponds to biotinylation patterns. DAPI was used to counterstain for nuclei. (**b**) Western Blot images of whole cell lysates of HEK293T cells 24h after transfection with indicated constructs either with or without biotin addition. Protein expression was detected with either anti-Flag or anti-FOXO1 antibodies. Biotinylation patterns were detected using Strep-HRP conjugate antibody. Anti-GAPDH or anti-betaTubulin antibodies were used for loading control. (**c**) Enrichment analysis for cellular component GO-terms of Strep-IP MS experiments performed using Metascape Software[91]. Analyzed protein lists were; significantly enriched proteins in N- or C-terminal BirA/PAX3-FOXO1 samples and top most frequent background proteins in BirA only samples detected at least in 2 out of 4 biological replicates (number of proteins indicated). Accession details for mass spectrometry datasets (PXD022187) can be found in the data availability section. (**d,e**) Validation of BAF complex members as potential proximal PAX3-FOXO1 candidates by BioID. Strep-IPs were performed 24h after transfection of HEK293T cells with indicated constructs either with or without biotin addition. Stainings were performed with indicated antibodies. PLK1 was used as a positive interaction control and several BAF complex members were validated. GAPDH and MYCN were used as cytoplasmic and nuclear controls. (**f**) Endogenous CoIP performed in RH4 (left) and RH30 (right) FP-RMS cell lines. PAX3-FOXO1 was immunoprecipitated using anti-FOXO1 antibody either with or without the addition of benzonase and empty beads were used as negative control. Immunoprecipitates were stained with indicated antibodies. GAPDH served as negative control. DNA digestion was analyzed by agarose gel electrophoresis.

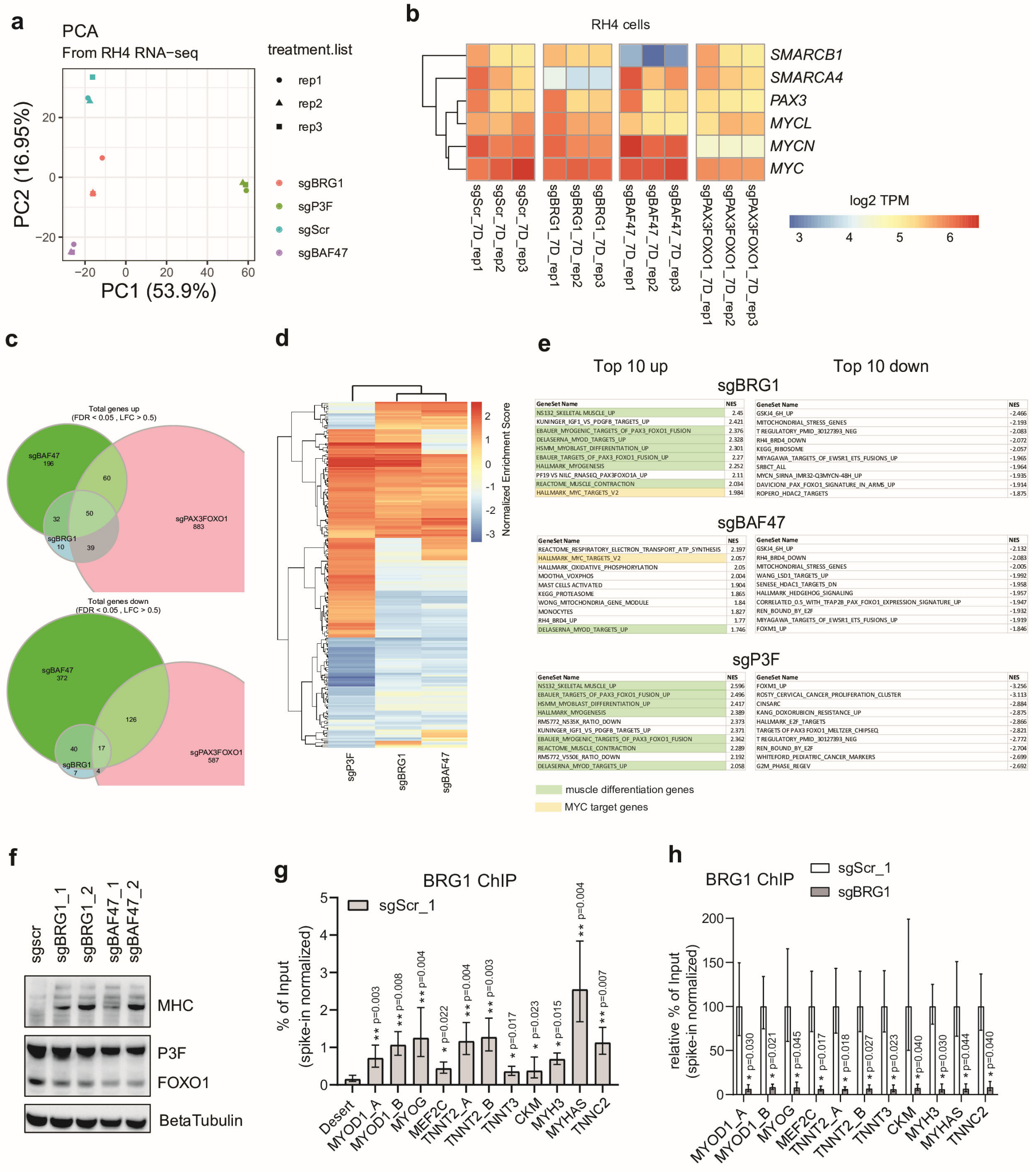

Figure S3. Induction of myogenic differentiation by interfering with BAF complex function. (**a**) Principal component analysis of RNA-seq experiments of Cas9 expressing RH4 cells 7 days after transduction with either guide RNAs against BRG1, SMARCB1 or PAX3-FOXO1 compared to negative control. (**b**) Expression level changes of various MYC isoforms as well as BRG1, SMARCB1 and PAX3 estimated in RNA-seq experiments (log2 TPM). (**c**) Differential gene expression analysis of Cas9 expressing RH4 cells 7 days after transduction with indicated sgRNAs compared to negative control. Numbers of up- and downregulated genes are indicated applying FDR < 0.05 and LFC > 0.5 thresholds. (**d**) Preranked GSEA heatmap depicting normalized enrichment scores including 513 different gene sets for the indicated sgRNAs 7d after transduction compared to negative control. (**e**) Top 10 up- and downregulated genesets as shown in (d) for each condition. (**f**) Western Blot images 7 days after transduction of Cas9 expressing RH4 cells with indicated sgRNAs. Upregulation of myosin heavy chains were confirmed on protein level after BRG1 or SMARCB1 targeting. (**g&h**) ChIP-qPCR assays with anti-BRG1 antibody performed for regions near differentiation target genes. 7 days after transduction of Cas9 expressing RH4 cells with indicated sgRNAs. (**g**) Recovery rates compared to negative control region under control conditions are depicted as mean and error bars indicating upper and lower limit values for 3 independent biological replicates. Statistical significance (based on dCt values) is given compared to desert region control by paired t-tests. (**h**) Relative recovery rates in BRG1 knockout cells compared to control conditions are depicted as mean and error bars indicating upper and lower limit values for 3 independent biological replicates. Statistical significance (based on dCt values) is given compared to negative control by paired t-tests (two-tailed). Accession details for RNA-seq and ChIP-seq data (GSE162052) can be found in the data availability section.

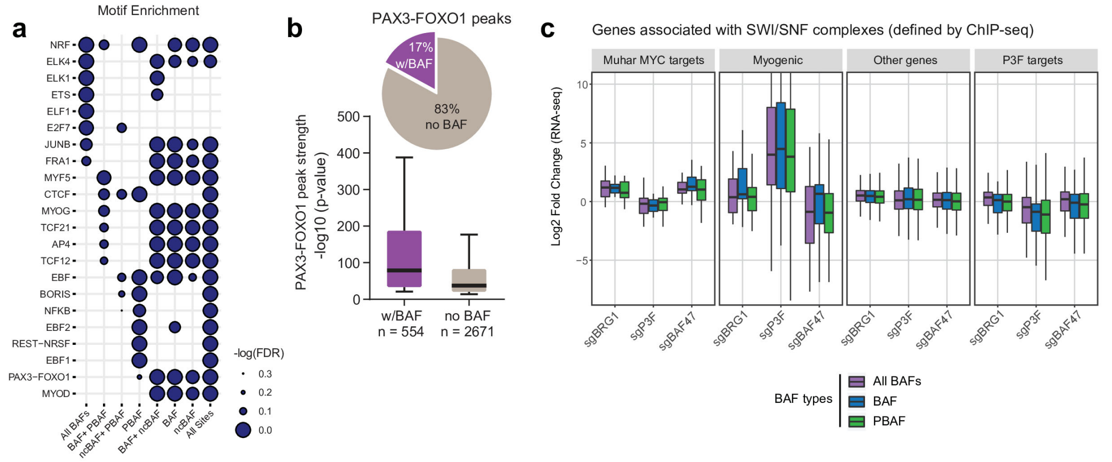

Figure S4. RMS-BAF complexes bind core regulatory circuitry (**a**) HOMER analysis from ChIP-seq experiments for sites occupied by BAF, PBAF, ncBAF complexes and combinations thereof. (**b**) PAX3-FOXO1 ChIP-seq peak strength in presence or absence of BAF complexes. Box plots of median and quartiles, with whiskers showing 1.5 × inter-quartile ranges. (**c**) Differential expression levels of gene sets associated with different mSWI/SNF subtype complexes in Cas9 expressing RH4 cells 7 days after transduction with indicated sgRNAs. Box plots of median and quartiles, with whiskers showing 1.5 × inter-quartile ranges. n=2 biologically independent RNA-seq samples and single ChIP-seq experiments. Accession details for RNA-seq and ChIP-seq data (GSE162052) can be found in the data availability section.

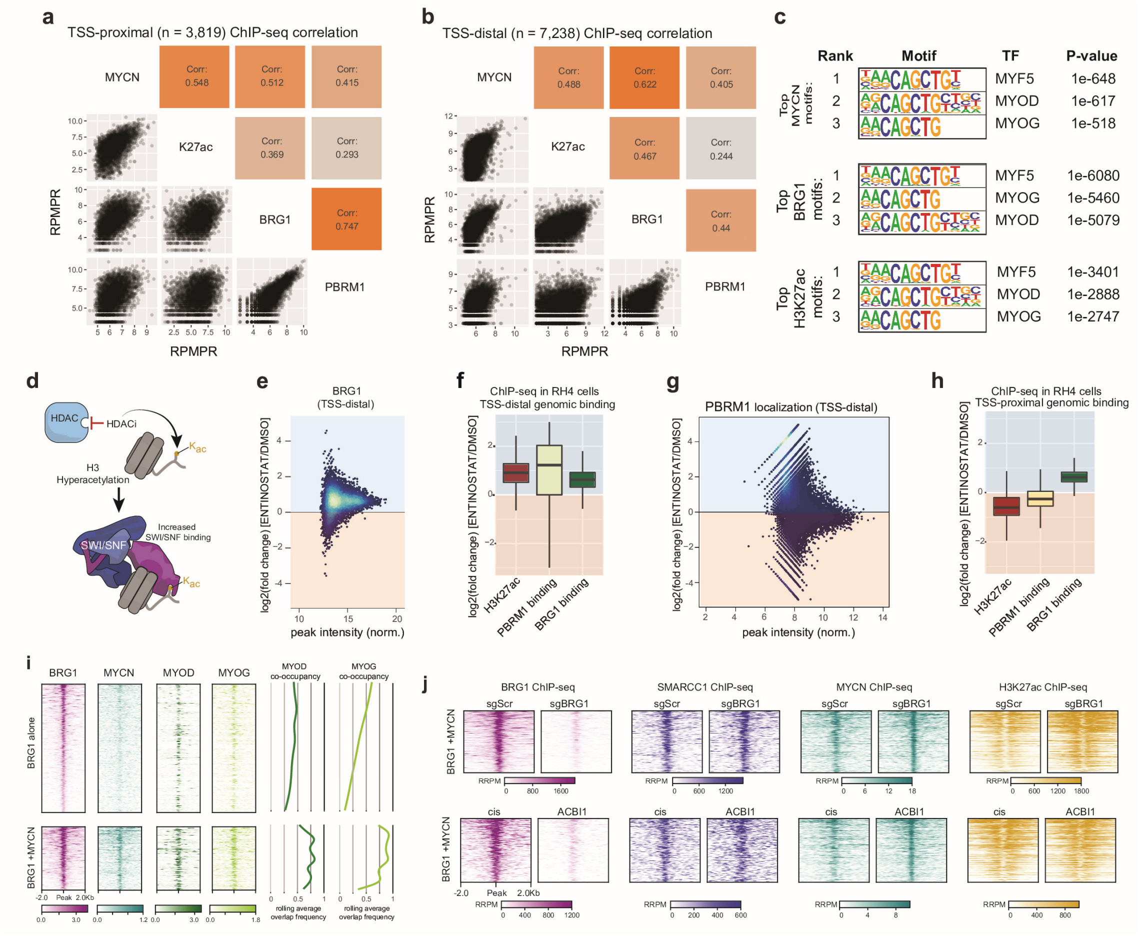

Figure S5. BAF complex senses H3K27ac status at enhancers. (**a**) Correlation analysis between MYCN, H3K27ac, BRG1 and PBRM1 genomic binding at TSS-proximal loci. (**b**) Correlation analysis between MYCN, H3K27ac, BRG1 and PBRM1 demonstrating positive association in all cases with strongest coefficient observed for MYCN and BRG1 binding. (**c**) HOMER analysis from ChIP-seq experiments for MYCN, BRG1 and H3K27ac, showing ranked motif enrichments. Ranked motifs are

shown (motif enrichment, y-axis), and labeled with defined consensus binding sites. Motif P-value statistics were performed using HOMER, which employs a ZOOP score (zero or one occurrence per sequence) coupled with the hypergeometric enrichment calculations (or binomial) to determine motif enrichment. This test is two-sided comparison of sequences in the called peaks to the genome-wide occurrence of those same sequences. (**d**) Proposed model for HDACi induced H3 hyperacetylation leading to increased SWI/SNF complex recruitment and binding. (**e**) ChIP-seq spike-in normalized MAPlot for BRG1 binding at TSS-distal loci as a function of 4h entinostat (2 μM) treatment. (**f**) Entinostat treatment induced changes of TSS-distal genomic deposition of H3K27ac and binding of PBRM1 as well as BRG1. Box plots of median and quartiles, with whiskers showing 1.5 × inter-quartile ranges. Data obtained from single experiments.(**g**) ChIP-seq spike-in normalized MAPlot for PBRM1 binding at TSS-distal loci as a function of 4h entinostat (2 μM) treatment. (**h**) Entinostat treatment induced changes of TSS-proximal genomic deposition of H3K27ac and binding of PBRM1 as well as BRG1. Box plots of median and quartiles, with whiskers showing 1.5 × inter-quartile ranges. Data obtained from single experiments. (**i**) CRTFs (MYCN, MYOG, MYOD1) co-occupy a subset of BRG1 sites in FP-RMS cells. Heatmaps show relative binding strength of indicated factors in BRG1 solo (top) and BRG1 + MYCN co-bound (bottom) regions. Right two panels display proportion of sites with overlapping MYOD1 and MYOG peaks reported as a rolling average across all sites. (**j**) Genetic removal of BRG1 in the top row, and chemical removal by ACBI1 in the bottom row, at MYCN co-occupied sites, showing removal of BRG1, stable binding of SMARCC1, increase in MYCN binding, and variable responses in H3K27ac. Accession details for ChIP-seq data (GSE162052) can be found in the data availability section.

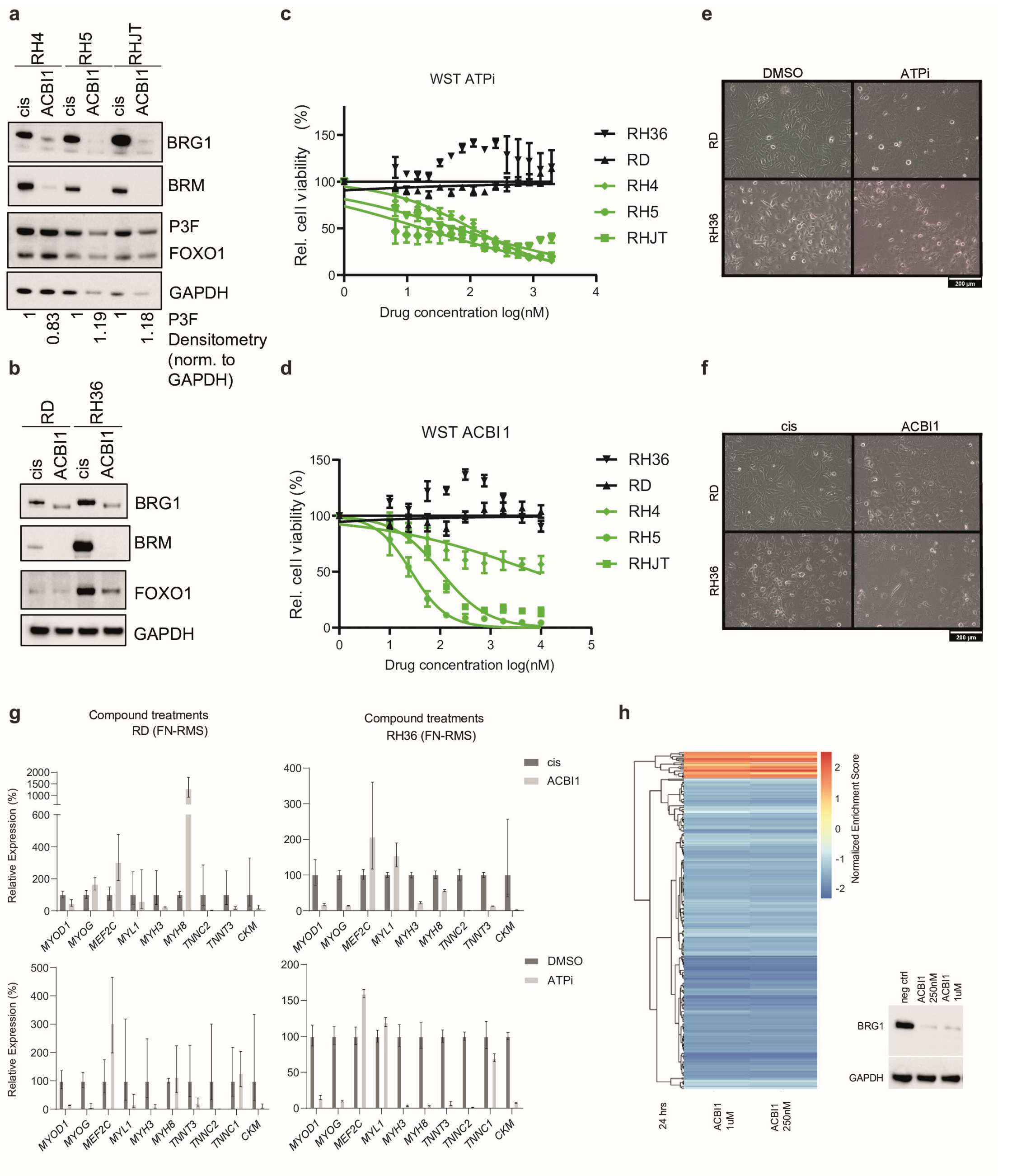

i

Top 10 down

GeneSet Name	NES	
	ACBI1_250nM_24h	ACBI1_1000nM_24h
DAVICIONI_TARGETS_OF_PAX_FOXO1_FUSIONS_UP	-2.245	-2.255
DAVICIONI_RHABDOMYOSARCOMA_PAX_FOXO1_FUSION_UP	-2.131	-2.059
MIYAGAWA_TARGETS_OF_EWSR1_ETS_FUSIONS_UP	-2.130	-2.167
RH4_BRD4_DOWN	-2.095	-2.110
PH1-LIKE UP	-2.077	-2.041
MCF10A_WT_P21KO_GENES_DESEQ_FINAL1_DOWN IN WT-SUPRESSED BY P21	-2.071	-2.201
HALLMARK_EPITHELIAL_MESENCHYMAL_TRANSITION	-2.068	-2.138
HENDRICKS_SMARCA4_TARGETS_UP	-2.053	-2.072
GRYDER_PAX3FOXO1_JQ1	-2.047	-2.082

PAX3-FOXO1 target genes
BRG1 target genes

Top 10 up

GeneSet Name	NES	
	ACBI1_250nM_24h	ACBI1_1000nM_24h
HALLMARK_MYC_TARGETS_V2	2.4172344	2.2299817
REACTOME_RESPIRATORY_ELECTRON_TRANSPORT_ATP_SYNTHESIS	2.311842	2.0445762
MOOTHA_VOXPHOS	2.1930246	2.1009338
HALLMARK_MYC_TARGETS_V1	1.9678597	1.9367707
CEGV2	1.9622833	1.7384866
DEPMAP_COMMON_ESSENTIALS	1.8819022	1.5548491
MITOCHONDRIA_PROTEOME_V1	1.7887365	1.5790097
WONG_MITOCHONDRIA_GENE_MODULE	1.7875595	1.6960089
KEGG_SPLICEOSOME	1.6648506	1.5488386

MYC target genes

Figure S6. Different effects of interference with SWI/SNF function in FP- and FN-RMS (**a&b**) Western blot images showing indicated protein levels in FP-RMS (RH4, RH5, RHJT) and FN-RMS (RD, RH36) cells treated with 250nM of PROTAC compound for 72h. Cis conformation compound was used as a negative control. (**c&d**) Cell viability as measured by WST-1 assay in different FP-RMS (RH4, RH5, RHJT) and FN-RMS (RD, RH36) cell lines treated with increasing concentrations of BRG1/BRM targeting ATPase inhibitor (ATPi) or PROTAC compound (ACBI1) for 72h normalized to DMSO control. Data points represent measurements of at least 4 technical replicates (mean values ±SD are shown). (**e&f**) Phase contrast images with inset scale bars in indicated FN-RMS cell lines treated with 3μM ATPase inhibitor (ATPi) or 250nM PROTAC (ACBI1) compared to control conditions**.** (**g**) Relative mRNA expression levels of muscle differentiation marker genes 72h after treatment of indicated FN-RMS cells with either PROTAC (ACBI1) or ATPase inhibitor (ATPi) compounds compared to negative control conditions measured by quantitative real-time PCR. Ct values relative to negative control treated cells were normalized to

GAPDH expression. Mean and error bars indicating upper and lower limit values are indicated for at least 3 independent biological replicates. (**h**) Pre-ranked GSEA heatmap depicting normalized enrichment scores including 513 different gene sets for the indicated treatment conditions compared to negative control for RH4 cells. Western Blot insert images are given to illustrate PROTAC efficiency at this timepoint (24 hours). (**i**) Top 10 up- and downregulated genesets as shown in (h) for each condition. Accession details for RNA-seq and ChIP-seq data (GSE162052) can be found in the data availability section.
