# Supplementary material for: BAF complexes drive proliferation and block myogenic differentiation in fusion-positive rhabdomyosarcoma
Source: Nat Commun. 2021 Nov 26;12:6924. doi: 10.1038/s41467-021-27176-w (PMC8626462; doi:10.1038/s41467-021-27176-w)
Supplement: Supplementary file 3 — Description of additional Supplementary File [file 41467_2021_27176_MOESM3_ESM.pdf]

Supplementary Data Legends

Supplementary Data S1:
Supplementary Data S1A. Results of EV2 and Royal chromatin regulatory domain CRISPR screens in RMS and non-RMS cells. Depletion values represent the $\log_2$(Fold Change) in guide RNA abundance at Day 12 vs. Day 3 for each RMS cell line as depicted in **Figure 1B**. Depletion values are averaged across multiple guides for each targeted domain. Depletion values in non-RMS cell lines represent the average across multiple control cell lines included in this study.
Supplementary Data S1B. Results of transcription factor CRISPR screen in RMS and non-RMS cells. Depletion values represent the $\log_2$(Fold Change) in guide RNA abundance at Day 12 vs. Day 3 for each RMS cell line as depicted in **Figure 1B**. Depletion values are averaged across multiple guides for each targeted protein. Depletion values in non-RMS cell lines represent the average across multiple control cell lines included in this study.
Supplementary Data S1C. Raw read counts for EV2 and Royal chromatin regulatory domain CRISPR screens in RMS cells.

Supplementary Data S2:
Supplementary Data S2A. Results of N-terminal PAX3-FOXO1 BioID experiments conducted in HEK293T cells. Results of MaxQuant analysis applying moderated t-test (for detailed information refer to methods section) are shown with key parameters including p-values and log2 FC values (complementing Figure 2a). Mass spectrometry was performed on Strep-IP samples after N-terminal BirA tagged PAX3-FOXO1 and BirA only overexpression of 4 biological replicates.
Supplementary Data S2B. Results of C-terminal PAX3-FOXO1 BioID experiments conducted in HEK293T cells. Results of MaxQuant analysis applying moderated t-test (for detailed information refer to methods section).are shown with key parameters including p-values and log2 FC values (complementing Figure 2b). Mass spectrometry was performed on Strep-IP samples after N-terminal BirA tagged PAX3-FOXO1 and BirA only overexpression of 4 biological replicates.

Supplementary Data S3:
Supplementary Data S3A. Proteomic characterization of fraction 16 collected from size-exclusion chromatography (SEC) of ammonium sulfate precipitated nuclear extracts from RH30 cells prior to immunoprecipitation. Peptide-spectrum match (PSM) values are provided for each identified protein.
Supplementary Data S3B. Proteomic characterization of fraction 17 collected from size exclusion chromatography (SEC) of ammonium sulfate precipitated nuclear extracts from RH30 cells prior to immunoprecipitation.
Supplementary Data S3C. Proteomic characterization of fraction 18 collected from size exclusion chromatography (SEC) of ammonium sulfate precipitated nuclear extracts from RH30 cells prior to immunoprecipitation.
Supplementary Data S3D. Proteomic characterization of fraction 16 collected from size exclusion chromatography (SEC) of ammonium sulfate precipitated nuclear extracts from RH4-P3F-FLAG cells prior to immunoprecipitation.

Supplementary Data S3E. Proteomic characterization of fraction 17 collected from size exclusion chromatography (SEC) of ammonium sulfate precipitated nuclear extracts from RH4-P3F-FLAG cells prior to immunoprecipitation.
Supplementary Data S3F. Proteomic characterization of fraction 18 collected from size exclusion chromatography (SEC) of ammonium sulfate precipitated nuclear extracts from RH4-P3F-FLAG cells prior to immunoprecipitation.

Supplementary Data S4:
Supplementary Data S4A. Proteins enriched by BRG1 immunoprecipitation of size-exclusion chromatography (SEC) fractions 16-18 from RH30 nuclear extracts. First biological replicate.
Supplementary Data S4B. Proteins enriched by BRG1 immunoprecipitation of SEC fractions 16-18 from RH30 nuclear extracts. Second biological replicate.
Supplementary Data S4C. Proteins enriched by BRG1 immunoprecipitation of SEC fractions 16-18 from RH4-P3F-FLAG nuclear extracts.
Supplementary Data S4D. Proteins enriched by IgG immunoprecipitation of SEC fractions 16-18 from RH30 nuclear extracts.
Supplementary Data S4E. Proteins enriched by BRM immunoprecipitation of SEC fractions 16-18 from RH30 nuclear extracts.
Supplementary Data S4F. Proteins enriched by PBRM1 immunoprecipitation of SEC fractions 16-18 from RH30 nuclear extracts.
Supplementary Data S4G. Proteins enriched by PBRM1 immunoprecipitation of SEC fractions 16-18 from RH4-P3F-FLAG nuclear extracts.

Supplementary Data S5:
Supplementary Data S5A. Plasmids and guide RNA sequences used in this study.
Supplementary Data S5B. Antibodies used in this study.
Supplementary Data S5C. TaqMan gene expression reagents used in this study.
Supplementary Data S5D. ChIP-qPCR and BioID cloning primer sequences.
Supplementary Data S5E. Compounds used in this study.
Supplementary Data S5F. Buffers and reagents used in this study.
Supplementary Data S5G. Software and algorithms used in this study.
