# Supplementary material for: BAF complexes drive proliferation and block myogenic differentiation in fusion-positive rhabdomyosarcoma
Source: Nat Commun. 2021 Nov 26;12:6924. doi: 10.1038/s41467-021-27176-w (PMC8626462; doi:10.1038/s41467-021-27176-w)
Supplement: Supplementary file 9 — Reporting Summary [file 41467_2021_27176_MOESM9_ESM.pdf]

nature research

Corresponding author(s): Benjamin Z. Stanton, Ph.D.
Beat Schäfer, Ph.D.
Javed Khan, M.D.

Last updated by author(s): 09/14/2021

Reporting Summary

Nature Research wishes to improve the reproducibility of the work that we publish. This form provides structure for consistency and transparency in reporting. For further information on Nature Research policies, see our Editorial Policies and the Editorial Policy Checklist.

Statistics

For all statistical analyses, confirm that the following items are present in the figure legend, table legend, main text, or Methods section.

n/a	Confirmed	
☐	☒	The exact sample size (*n*) for each experimental group/condition, given as a discrete number and unit of measurement
☐	☒	A statement on whether measurements were taken from distinct samples or whether the same sample was measured repeatedly
☐	☒	The statistical test(s) used AND whether they are one- or two-sided<br>*Only common tests should be described solely by name; describe more complex techniques in the Methods section.*
☐	☒	A description of all covariates tested
☐	☒	A description of any assumptions or corrections, such as tests of normality and adjustment for multiple comparisons
☐	☒	A full description of the statistical parameters including central tendency (e.g. means) or other basic estimates (e.g. regression coefficient) AND variation (e.g. standard deviation) or associated estimates of uncertainty (e.g. confidence intervals)
☐	☒	For null hypothesis testing, the test statistic (e.g. *F*, *t*, *r*) with confidence intervals, effect sizes, degrees of freedom and *P* value noted<br>*Give P values as exact values whenever suitable.*
☒	☐	For Bayesian analysis, information on the choice of priors and Markov chain Monte Carlo settings
☒	☐	For hierarchical and complex designs, identification of the appropriate level for tests and full reporting of outcomes
☒	☐	Estimates of effect sizes (e.g. Cohen's *d*, Pearson's *r*), indicating how they were calculated

Our web collection on statistics for biologists contains articles on many of the points above.

Software and code

Policy information about availability of computer code

Data collection	BioRad Image Lab 5.2.1 (Build 11) was used for chemiluminescence detection of Western Blots.<br>BioTek Gen5 2.07.17 was used for measurement of colorimetric assays (WST).<br>BD LSR Fortessa Cell Analyzer was used to measure flow cytometry data.<br>Olympus CKX41 Inverted Microscope was used for phase contrast image aquisition.<br>Nikon Eclipse Ti2 was used for Immunofluorescence microscopy.<br>Thermo Scientific Orbitrap Fusion Tribrid Mass Spectrometer was used for measurements in BioID experiments.<br>Illumina NovaSeq 6000 system was used for RNA-Sequencing (performed by Atlas Biolab GmBH, Berlin, Germany)<br>Illumina NextSeq 500 system was used for ChIP-Seq experiments.<br>ABI 7900UT Fast Real-Time PCR System was used for RT-qPCR data aquisition.
Data analysis	Prism 8.0.0 (from GraphPad Software), R (version 3.5.1 from the Foundation for Statistical Computing), or GSEA (version 4.0 from the Broad Institute) were used for statistical analyseslowJo V10.6.1 software was used to analyze flow cyt<br>Lumenera Infinity capture software (V5.0.2) was used to process phase contrast images.<br>Nikon NIS-Elements AR5.21.02 (Build 1487) software was used to process immunofluorescence pictures.<br>ABI SDS 2.4 software was used for RT-qPCR data processing.<br>Protein identification and quantification of BioID experiments were performed using MaxQuant software and Andromeda<br>search engine (see Methods section for detailed information). Gene ontology enrichment analysis was performed using Metascape software (https://metascape.org/gp/index.html#/main/step1)<br>A custom code for RNA-seq and ChIP-seq analyses is available on github at https://github.com/GryderArt.<br>BCHNV software: https://github.com/CBIIT/ChIP_seq/blob/master/scripts/runBCHNV.sh<br>CLTRON software: https://pypi.org/project/coltron/<br>DESeq2 (1.28.1) was used in differential gene analysis.

RNA-seq reads were mapped to the human genome build hg19 by STAR (https://github.com/alexdobin/STAR) and quantified using RSEM (https://deweylab.github.io/RSEM/). Gene set enrichment was assessed using GSEA software (https://www.gsea-msigdb.org/gsea/) and visualized in R (https://github.com/GryderArt/VisualizeRNAseq).
ChIP-seq data was mapped to hg19 using BWA. For ChIP-Rx, we additionally mapped spike in reads to dm3 using BWA (version 0.7.17), and normalized human reads to million-mapped Drosophila reads (RRPM, reference normalize reads per million). Peaks were called using MACS2.0, with stringency thresholds of p = 0.0000001 and filtered to remove ENCODE blacklisted regions (satellite repeats and spuriously over-mapped regions). Peak intersections we identified using bedtools intersect, and the resulting heatmaps and metagene plots were plotted using deeptools (https://deeptools.readthedocs.io/en/develop/). Genome tracks were visualized in IGV.
For Mass Spectrometry data collection, peak lists were generated using FCC and Proteome Discoverer2.4.
BioID experiments were quantified by MaxQuant version 1.6.2.3.

For manuscripts utilizing custom algorithms or software that are central to the research but not yet described in published literature, software must be made available to editors and reviewers. We strongly encourage code deposition in a community repository (e.g. GitHub). See the Nature Research guidelines for submitting code & software for further information.

Data

Policy information about availability of data

All manuscripts must include a data availability statement. This statement should provide the following information, where applicable:
- Accession codes, unique identifiers, or web links for publicly available datasets
- A list of figures that have associated raw data
- A description of any restrictions on data availability

For ChIP-Seq data analysis we used the publicly available ENCODE Consortium (https://sites.google.com/site/ anshulkundaje/projects/blacklists) dataset, as well as two ChIP-seq datasets recently reported (GSE116344, GSE83725; GEO Omnibus) in Fig.4. Additionally, we used gene expression datasets (phs000720 dbGAP) for comparing expression levels of mSWI/SNF subunits in RMS vs. normal muscle tissue for Fig. S1B. In addition we used publicly available cancer dependency datasets (https://depmap.org/portal/) to illustrate RMS cell dependency on BRG1 (SMARCA4) compared to other cancer types.
The data sets of RNA-seq and ChIP-seq generated in this study have been deposited in the Gene Expression Omnibus database with accession number GSE162052. The mass spectrometry proteomics data of our BioID experiments supporting the findings in Figure 2A,B have been deposited to the ProteomeXchange Consortium via the PRIDE90 partner repository with the dataset identifier PXD022187. The mass spectrometry proteomics data of SEC-IP-MS experiments supporting the findings in Figure 2F-H (2F:6LTJ, 2G:6TDA) have been made publicly available and were deposited to the ProteomeXchange Consortium via the MassIVE partner repository with the dataset identifier MSV000086494.

For filtering ChIP-Seq Peaks (Fig. 3E-G, Fig. 4, Fig. 5, Fig. S4, Fig. S5) we used blacklist dataset from the ENCODE Consortium (https://sites.google.com/site/ anshulkundaje/projects/blacklists).
Mass spectrometry proteomic data of BioID experiments (Fig. 2A/B, Fig. S2C, Table S2) are available via PRIDE repository with the identifier PXD022187 (Reviewer account details: Username: reviewer_pxd022187@ebi.ac.uk Password: U0zrLLaH)
Mass spectrometry proteomic data of SEC-IP experiments (Fig. 2F-H) are deposited to the ProteomeXchange Consortium via the MassIVE partner repository with the dataset identifier MSV000086494. (publicly available)
The data sets of RNA-seq (Fig. 3C/E-G, Fig. 4G, Fig. S3A/B, Fig. S4C, Fig. S6h,i) and ChIP-seq (Fig. 3E-G, Fig. 4, Fig. 5, Fig. S4, Fig. S5, Fig.S6) generated in this study have been deposited in the Gene Expression Omnibus database with accession number GSE162052.
A Source Data file is available containing all relevant associated raw data.

Field-specific reporting

Please select the one below that is the best fit for your research. If you are not sure, read the appropriate sections before making your selection.

☒ Life sciences ☐ Behavioural & social sciences ☐ Ecological, evolutionary & environmental sciences

For a reference copy of the document with all sections, see nature.com/documents/nr-reporting-summary-flat.pdf

Life sciences study design

All studies must disclose on these points even when the disclosure is negative.

Sample size	No statistical method for sample size calculations were performed. We have used minimal sample size sufficient to detect biological differences. Numbers of samples are indicated in the figure legends for each panel.
Data exclusions	Outliers found in technical replicates of Ct-threshold measurements within RT-qPCR experiments (pre-established SD > 0.5) were removed from the analysis.<br>Single measurements of technical replicates within WST-1 measurements had to be excluded due to pipetting errors (see source data file).<br>Otherwise no data was excluded.
Replication	All experiments were performed at least twice, in most cases more than this. All replicates were succesful and are included.<br>Number of biological replicates are given where applicable.<br>ChIP-Seq experiments were performed as single replicates.
Randomization	Our experiments were not randomized, because we have controls run side-by-side with the experimental samples.

Blinding	Our experiments were not blinded, because we have controls run side-by-side with the experimental samples.

Reporting for specific materials, systems and methods

We require information from authors about some types of materials, experimental systems and methods used in many studies. Here, indicate whether each material, system or method listed is relevant to your study. If you are not sure if a list item applies to your research, read the appropriate section before selecting a response.

Materials & experimental systems

n/a	Involved in the study
☐	☒ Antibodies
☐	☒ Eukaryotic cell lines
☒	☐ Palaeontology and archaeology
☒	☐ Animals and other organisms
☒	☐ Human research participants
☒	☐ Clinical data
☒	☐ Dual use research of concern

Methods

n/a	Involved in the study
☐	☒ ChIP-seq
☐	☒ Flow cytometry
☒	☐ MRI-based neuroimaging

Antibodies

Antibodies used

For Western Blot:
beta Tubulin (D309W), 86298S, Cell Signaling Technologies. Used as 1/1000 dilution.
BRG1 (A52), 3508S, Cell Signaling Technologies. Used as 1/500 dilution.
BRM (D9E8B), 11966S, Cell Signaling Technologies. Used as 1/1000 dilution.
Cleaved Caspase 7 (D198), 9491S, Cell Signaling Technologies. Used as 1/1000 dilution.
Flag (clone M2), F1804, Sigma Aldrich. Used as 1/1000 dilution.
FOXO1 (H-128), sc-11350, Santa Cruz Biotechnologies. Used as 1/500 dilution.
GAPDH (14C10), 2118L, Cell Signaling Technologies. Used as 1/1000 dilution.
MYHC, MF20-c, Developmental Studies Hybridoma Bank. Used as 1/500 dilution.
MYCN, 9405S, Cell Signaling Technologies. Used as 1/1000 dilution.
PARP (46D11), 9532, Cell Signaling Technologies. Used as 1/1000 dilution.
PLK1 (clone 35-206), 05-844, Millipore. Used as 1/500 dilution.
SMARCB1/BAF47 (D8M1X), 91735S, Cell Signaling Technologies. Used as 1/1000 dilution.
SMARCC1/BAF155 (D7F8S), 11956S, Cell Signaling Technologies. Used as 1/1000 dilution.
Strep-HRP, 016-030-084, Jackson Immunoresearch. Used as 1/500 dilution.
anti-mouse HRP, 7076, Cell Signaling Technologies. Used as 1/2000 dilution.
anti-rabbit HRP, 7074, Cell Signaling Technologies. Used as 1/2000 dilution.
easyBlot anti-mouse HRP, GTX221667-01, GeneTex. Used as 1/1000 dilution.
easyBlot anti-rabbit HRP, GTX221666-01, GeneTex. Used as 1/1000 dilution.
PBRM1 (BAF180), 91894, Cell Signaling Technologies. Used as 1/1000 dilution.
BAF47 (SMARCB1), ab192864, Abcam. Used as 1/1000 dilution.
SMARCC2 (D8O9V), 12760S, Cell Signaling Technologies. Used as 1/1000 dilution.
SMARCE1 (E6H5J), 33360S, Cell Signaling Technologies. Used as 1/1000 dilution.
BRD9, ab137245, Abcam. Used as 1/1000 dilution.
DPF2, ab134942, Abcam. Used as 1/1000 dilution.

For SEC-IP-MS:
IgG, I5006, Sigma. Used as 3ug in 1 ml dilution.
PBRM1 (BAF180), 91894, Cell Signaling Technologies. Used as 3ug in 1 ml dilution.
BRG1, ab110641, Abcam. Used as 3ug in 1 ml dilution.

For CoIP:
BRG1, ab110641, Abcam. Used as 4ug in 2 ml dilution.
FOXO1 (C-20), sc-9808, Santa Cruz Biotechnologies. Used as 8ug in 2 ml dilution.

For Immunofluorescence:
Flag, F1804, Sigma Aldrich. Used as 1/500 dilution.
Strep Alexa-Fluor 594 conjugate, S11227, Thermo Fisher. Used as 1/250 dilution.
MHC, MF20-c, Developmental Studies Hybridoma Bank. Used as 1/50 dilution.
Alexa-Fluor 488 goat anti-mouse, A11029, Thermo Fisher. Used as 1/250 dilution.
Alexa-Fluor 594 goat anti-mouse, A11032, Thermo Fisher. Used as 1/250 dilution.

For ChIP:

BRG1, ab110641, Abcam: used 4 ug antibody in 350uL reaction volume.
H3K27ac, 39133, Active Motif: used 4 ug antibody in 350uL reaction volume.
MYCN (NCM-II 100), MA1-170, Thermo Fisher: used 7 ug antibody in 350uL reaction volume.
SMARCC1, ab172638, Abcam: used 4 ug antibody in 350uL reaction volume.
Drosophila Spike-in Antibody, 61686, Active Motif: used 2 ug antibody in 350uL reaction volume (for 25 ug of chromatin sample).
BRD9, ab137245, Abcam: used 4 ug antibody in 700uL reaction volume.
DPF2, ab134942, Abcam: used 4 ug antibody in 700uL reaction volume.
PBRM1, 61381, Active Motif: used 4 ug antibody in 700uL reaction volume.

Validation

Validation data for the application Western Blot for all the following antibodies from CellSignaling is available on the CellSignaling homepage:
beta Tubulin (D309W), 86298S, Cell Signaling Technologies: β-Tubulin (D3U1W) Mouse mAb recognizes endogenous levels of total β-tubulin protein, and reactive to human, mouse, rat, hamster, and monkey β-Tubulin.
BRG1 (A52), 3508S, Cell Signaling Technologie: Brg1 (A52) Antibody detects endogenous levels of Brg1 protein in human, mouse and Monkey.
BRM (D9E8B), 11966S, Cell Signaling Technologies: BRM (D9E8B) XP® Rabbit mAb recognizes endogenous levels of total BRM protein in human and Monkey. This antibody does not cross-react with BRG1 protein.
Cleaved Caspase 7 (D198), 9491S, Cell Signaling Technologies: Cleaved Caspase-7 (Asp198) Antibody detects endogenous levels of the large fragment of caspase-7 resulting from cleavage at aspartic acid 198 for human, mouse, rat, and Monkey. The antibody does not cross-react with full length caspase-7 or with other caspases.
GAPDH (14C10), 2118L, Cell Signaling Technologies: GAPDH (14C10) Rabbit mAb detects endogenous levels of total GAPDH protein in human, mouse, rat, Monkey, Bobine, and pig.
MYCN, 9405S, Cell Signaling Technologies: N-Myc Antibody detects endogenous levels human N-Myc . It does not cross-react with other Myc family members.
PARP (46D11), 9532, Cell Signaling Technologies: PARP (46D11) Rabbit mAb detects endogenous levels of total full-length PARP-1 and the large fragment (89 kDa) produced by caspase cleavage at Asp214 in human, mouse, rat and Monkey. This antibody does not cross-react with PARP-2 and PARP-3.
SMARCB1/BAF47 (D8M1X), 91735S, Cell Signaling Technologies:SMARCB1/BAF47 (D8M1X) Rabbit mAb recognizes endogenous levels of total SMARCB1/BAF47 protein in human, mouse, rat, and monkey.
SMARCC1/BAF155 (D7F8S), 11956S, Cell Signaling Technologies: SMARCC1/BAF155 (D7F8S) Rabbit mAb recognizes endogenous levels of total SMARCC1/BAF155 protein in human, mouse, rat, and monkey.
anti-mouse HRP, 7076, Cell Signaling Technologies: horse anti-mouse IgG, HRP-linked antibody
anti-rabbit HRP, 7074, Cell Signaling Technologies: goat anti-rabbit IgG, HRP-linked Antibody
PBRM1 (BAF180), 91894, Cell Signaling Technologies: PBRM1 (D3F7O) Rabbit mAb recognizes endogenous levels of total PBRM1 protein in human, mouse, rat and monkey.
PLK1 (clone 35-206), 05-844, Millipore, was used in numerous publications for Western Blot listed on the manufacturers website (https://www.emdmillipore.com/US/en/product/Anti-PLK1-Antibody-clone-35-206,MM_NF-05-844?ReferrerURL=https%3A%2F%2Fwww.google.com%2F).
FOXO1 (H-128; Santa Cruz Biotechnology sc-11350) was used in numerous publications for Western Blot listed on the SantaCruz website (https://www.scbt.com/p/fkhr-antibody-h-128)
MHC, MF-20 c, Developmental Studies Hybridoma bank, was used in numerous publications for Western Blot listed on the Developmental Studies Hybridoma Bank website (https://dshb.biology.uiowa.edu/MF-20)
For BAF47 (SMARCB1), ab192864, Abcam, validation data for Western Blot is available on the manufacturers website. Abpromise guarantee this product for flow cytometry, IHC-P, IP, and WB.

Antibodies used for Immunoprecipitation:
For BRG1, ab110641, Abcam, numerous publications for Immunopecipitation and ChIP are listed on the manufacturers homepage.
For PBRM1 (BAF180), 91894, Cell Signaling Technologies, validation data for Immunoprecipitation is available on the manufacturers website
The use of FOXO1 (C-20), sc-9808, Santa Cruz Biotechnologies, for CoIP has been validated by the group of authors (Böhm, JCI, 2016)

Validation data for the application ChIP for all the following antibodies from Active Motif is available on the CellSignaling homepage:
H3K27ac, 39133, Active Motif
Drosophila Spike-in Antibody, 61686, Active Motif
PBRM1, 61381, Active Motif

For SMARCC1, ab172638, Abcam, validation data for the application ChIP is available on the manufacturers website

The following antibodies from Abcam have been used for ChIP in publications listed on the manufacturers website
BRG1, ab110641, Abcam
BRD9, ab137245, Abcam
DPF2, ab134942, Abcam

MYCN (NCM-II 100), MA1-170, Thermo Fisher has been chosen based on publications using the same clone by different vendors (Abcam, publications listed on webpage) for its superior concentration. Results using this antibody were validated using previously described MYCN binding sites in RMS cells (Gryder, Cancer Discovery, 2017) by ChIP-qPCR.

MHC, MF-20 c, Developmental Studies Hybridoma bank, was used in numerous publications for immunofluorescence listed on the Developmental Studies Hybridoma Bank website.

Eukaryotic cell lines

Policy information about cell lines

Cell line source(s)	The cell lines RH4, RHJT, RH36 ere all provided by Peter Houghton, Greehey Children's Cancer Research Institute, San Antonio, Texas, USA. RH5 cells were provided by Susan Ragsdale, St. Jude Children's Hospital, Memphis, TN. RH30, RD as well as HEK293T cells were purchased through ATCC. SMS-CTR cells were originally established by Patrick Reynolds, University of Texas Health Science Center, Dallas, Texas.
Authentication	All cell lines were authenticated by short tandem repeat analysis (STR profiling) and positively matched with reference data.
Mycoplasma contamination	Cell lines were regularly tested for Mycoplasma and were Mycoplasma free.
Commonly misidentified lines (See ICLAC register)	None as far as we know.

ChIP-seq

Data deposition

☒ Confirm that both raw and final processed data have been deposited in a public database such as GEO.

☒ Confirm that you have deposited or provided access to graph files (e.g. BED files) for the called peaks.

Data access links *May remain private before publication.*	ChIP-seq data generated in this study have been deposited in the Gene Expression Omnibus database with accession number GSE162052.
Files in database submission	Raw sequencing filess (.fastq) and peak files (.bed) are available for the following samples:<br>RH4_DMSO_4h_BRG1<br>RH4_Ent_4h_BRG1<br>RH4_DMSO_4h_PBRM1<br>RH4_Ent_4h_PBRM1<br>RH4_sgScr_input<br>RH4_sgScr_BRG1<br>RH4_sgScr_HDAC2<br>RH4_sgScr_H3K27ac<br>RH4_sgScr_H3K9ac<br>RH4_sgBRG1_input<br>RH4_sgBRG1_BRG1<br>RH4_sgBRG1_HDAC2<br>RH4_sgBRG1_H3K27ac<br>RH4_sgBRG1_H3K9ac<br>RH4_BRD9<br>RH4_DPF2<br>RH4_cis_7d_BRG1<br>RH4_cis_7d_SMARCC1<br>RH4_cis_7d_H3K27ac<br>RH4_ACBI1_7d_BRG1<br>RH4_ACBI1_7d_SMARCC1<br>RH4_ACBI1_7d_H3K27ac<br>RH4_sgScr_SMARCC1<br>RH4_sgBRG1_SMARCC1<br>RH4_cis_7d_MYCN_r2<br>RH4_ACBI1_7d_MYCN_r2<br>RH4_sgScr_MYCN_r2<br>RH4_sgBRG1_MYCN_r2
Genome browser session (e.g. UCSC)	No longer applicable.

Methodology

Replicates	Experiments were performed as single replicates

Sequencing depth

All samples were sequenced with at total read cound of 25-30 millions.
Readlength in all samples was 75bp and sequencing was performed as single-end.

Antibodies

BRG1, ab110641, Abcam
H3K27ac, 39133, Active Motif
H3K9ac, Cell Signaling Technologies, 9649
HDAC2, Abcam, ab7029
MYCN (NCM-II 100), MA1-170, Thermo Fisher
SMARCC1, ab172638, Abcam
Drosophila Spike-in Antibody, 61686, Active Motif
BRD9, ab137245, Abcam
DPF2, ab134942, Abcam
PBRM1, 61381, Active Motif

Peak calling parameters

ChIP-seq data was mapped to hg19 using BWA. For ChIP-Rx, we additionally mapped spike in reads to dm3 using BWA, and normalized human reads to million-mapped Drosophila reads (RRPM, reference normalize reads per million). Peaks were called using MACS2.0, with stringency thresholds of p = 0.0000001 and filtered to remove ENCODE blacklisted regions (satellite repeats and spuriously over-mapped regions). Peak intersections we identified using bedtools intersect, and the resulting heatmaps and metagene plots were plotted using deeptools (https://deeptools.readthedocs.io/en/develop/). Genome tracks were visualized in IGV.

Data quality

SampleFile PEAKS_PVALUE7 PEAKS_FOLD_CHANGE5
Sample_RH4_DMSO_4h_BRG1_040_C_HKLNMBGX9 15991 14811
Sample_RH4_Ent_4h_BRG1_040_C_HKLNMBGX9 25445 23976
Sample_RH4_DMSO_4h_PBRM1_040_C_HKLNMBGX9 19837 19376
Sample_RH4_Ent_4h_PBRM1_040_C_HMFTFBGX7 43050 42106
Sample_RH4_sgScr_BRG1_C_H7HJ5BGXC 54372 49046
Sample_RH4_sgScr_HDAC2_C_H7HJ5BGXC 42691 36683
Sample_RH4_sgScr_H3K27ac_C_H7HJ5BGXC 55373 34389
Sample_RH4_sgScr_H3K9ac_C_H7JYLBGXC 44138 40106
Sample_RH4_sgBRG1_BRG1_C_H7JYLBGXC 16089 14506
Sample_RH4_sgBRG1_HDAC2_C_H7JYLBGXC 4726 3741
Sample_RH4_sgBRG1_H3K27ac_C_H7JYLBGXC 60474 55872
Sample_RH4_sgBRG1_H3K9ac_C_H7JYLBGXC 48605 44026
Sample_RH4_BRD9_200413_C_BZS 21092 18179
Sample_RH4_DPF2_200413_C_BZS 23786 19840
Sample_RH4_cis_7d_BRG1_C_HM35YBGXF 209485 196812
Sample_RH4_cis_7d_SMARCC1_C_HM35YBGXF 59907 57914
Sample_RH4_cis_7d_H3K27ac_C_HM35YBGXF 50960 47669
Sample_RH4_ACBI1_7d_BRG1_C_HM35YBGXF 503834 468047
Sample_RH4_ACBI1_7d_SMARCC1_C_HM35YBGXF 98672 94203
Sample_RH4_ACBI1_7d_H3K27ac_C_HM35YBGXF 43674 40961
Sample_RH4_sgScr_SMARCC1_C_HM35YBGXF 71696 69184
Sample_RH4_sgBRG1_SMARCC1_C_HM35YBGXF 61163 59035
Sample_RH4_cis_7d_MYCN_r2_C_HM35YBGXF 2567 2119
Sample_RH4_ACBI1_7d_MYCN_r2_C_HM35YBGXF 1958 1595
Sample_RH4_sgScr_MYCN_r2_C_HM35YBGXF 802 653
Sample_RH4_sgBRG1_MYCN_r2_C_HM35YBGXF 3167 2683

Software

ChIP-seq data was mapped to hg19 using BWA. For ChIP-Rx, we additionally mapped spike in reads to dm3 using BWA, and normalized human reads to million-mapped Drosophila reads (RRPM, reference normalize reads per million). Peaks were called using MACS2.0, with stringency thresholds of p = 0.0000001 and filtered to remove ENCODE blacklisted regions (satellite repeats and spuriously over-mapped regions). Peak intersections we identified using bedtools intersect, and the resulting heatmaps and metagene plots were plotted using deeptools (https://deeptools.readthedocs.io/en/develop/). Genome tracks were visualized in IGV.

Flow Cytometry

Plots

Confirm that:

- [x] The axis labels state the marker and fluorochrome used (e.g. CD4-FITC).
- [x] The axis scales are clearly visible. Include numbers along axes only for bottom left plot of group (a 'group' is an analysis of identical markers).
- [x] All plots are contour plots with outliers or pseudocolor plots.
- [x] A numerical value for number of cells or percentage (with statistics) is provided.

Methodology

Sample preparation	All flow cytometry experiments were performed on cell lines.<br>For competition assays, Cas9 expressing RH4 cells (GFP positive) were plated in 24-well format and transduced the next day with sgRNA carrying lentiviruses (with RFP reporter). 2 days after transduction, transduced cells were mixed with same amount of untransduced cells. Part of the resulting mixture was directly used for baseline flow cytometry measurements, while the rest was kept in culture. For flow cytometry analysis, cells were fixed with 0.5% Paraformaldehyde/1xPBS and washed twice in 1xPBS. After resuspension of cell pellets in 1xPBS, samples were analyzed.<br>For cell cycle analysis, Cas9 expressing RH4 cells were transduced with indicated sgRNAs. Cells were harvested by trypsinization, washed in 1xPBS, fixed in 70% ice-cold Ethanol and incubated at -20°C for at least 2 hours. Before flow cytometry, cells were washed by PBS and resuspended in 500µl PI solution (Table S5F). Data were processed by FlowJo V10.6.1 software using Dean-Jett-Fox model to assign cell cycle phases.
Instrument	Flow cytometry data was collected with BD LRS Fortessa Cell Analyzer
Software	Flow cytometry data was analyzed using BD FlowJo V10.6.1 software
Cell population abundance	Sorted RH4 Cas9 NG cells were regularly checked for GFP positivity compared to RH4 WT cells, which remained stable over time (>95% positive).
Gating strategy	In all experiments, gating was performed to include live cells only (SSC-A/FSC-A). Of this population, single cells were extracted (FSC-H/FSC-A). According to the different experiments, BFP, RFP and PI signal intensities were determined. For boundary determination using fluorescent proteins, gating was adjusted according to untransduced cells. For detailed information see Source Data file.

- [x] Tick this box to confirm that a figure exemplifying the gating strategy is provided in the Supplementary Information.
